# Unexplained persistent postpartum palpitations and tachycardia due to Group A Streptococcus

**DOI:** 10.1186/s13104-015-1739-y

**Published:** 2015-11-30

**Authors:** Nathan A. Keller, Xin Guan, Alicia Wiczulis, Paul Burcher

**Affiliations:** Department of Obstetrics and Gynecology, Albany Medical Center, 16 New Scotland Avenue, Second Floor, MC-74, Albany, NY 12208 USA; Albany Medical College, 43 New Scotland Avenue, Albany, NY 12208 USA

**Keywords:** Group A Streptococcus, Toxic shock syndrome, Septic shock, Compartment syndrome, Persistent postpartum tachycardia, Palpitations

## Abstract

**Background:**

Group A Streptococcus is one of the most morbid infections in modern obstetric practice. Pregnant women are known to have a 20-fold increased risk of invasive Group A Streptococcus with greatest risk in the first 4 days postpartum. The overwhelming majority of these infections will present with fever, uterine tenderness, or vaginal discharge. A much smaller subset may present to the Emergency Room after initial hospital discharge with much less obvious symptoms. In our case, persistent palpitations with unexplained tachycardia led to improper diagnosis in multiple Emergency Rooms.

**Case presentation:**

A 37 year-old Caucasian female presents with four post-partum days of unexplained sinus tachycardia and absence of fever, uterine tenderness, or vaginal discharge, which elicits an extensive cardiac and pulmonary workup in multiple Emergency Rooms. Consequent late diagnosis of invasive Group A Streptococcus infection lead to significantly increased morbidity including toxic shock syndrome, acute renal failure, total abdominal hysterectomy and bilateral salpingo-oophorectomy, multiple laparotomies, fasciotomy, intubation, continuous renal replacement therapy, and extensive hospital course and recovery.

**Conclusion:**

Persistent palpitations with unexplained tachycardia in the post-partum patient in the Emergency Room setting is a potential early warning of Group A Streptococcus infection. Even in the absence of reported clinical fever, uterine tenderness, or vaginal discharge, an early speculum and pelvic exam, with or without consultation with the obstetrics service, is prudent due to the potentially high morbidity or even fatality of Group A Streptococcus infection.

## Background

The incidence of puerperal Group A Streptococcus (GAS) infection is 6 per 100,000 live births in the United States [1]. Pregnant and postpartum women have a 20-fold increased risk of invasive GAS infection compared with non-pregnant women [2]. The incidence of epidemic puerperal sepsis due to GAS was reduced with the introduction of hand washing, antiseptics and penicillin [3]. However, invasive GAS infection associated with pregnancy and childbirth reemerged in the mid-1980s and is associated with high morbidity and mortality compared with other maternal infections [4]. Transmission can occur from healthcare providers, other patients, or a community-acquired source [5]. About 14 % of puerperal GAS infections are nosocomially acquired [6]. It is transmitted by respiratory droplets or contact with secretions from lesions. Infections occur predominantly in the postpartum period within the first 4 days and maternal mortality is the highest in the first 2 days [6]. Mortality approaches 60 % when shock develops [7]. Currently, there is no screening process for GAS in the postpartum period and no algorithm for treatment.

Invasive GAS infection can cause endometritis, necrotizing fasciitis of the reproductive organs, and if severe, toxic shock syndrome. Rarely, it can cause non-gynecologic infections such as empyema, necrotizing fasciitis of the extremities, and pyomyositis. Necrotizing soft tissue infections in the obstetrical population are most commonly associated with GAS and can range from cellulitis, to necrotizing fasciitis, to death from septic shock [8]. Cellulitis is an infection of the dermis and subcutaneous tissue. The tissue is usually erythematous, tender and warm with the patient presenting with fever and chills [5]. The first symptom of necrotizing fasciitis is usually diffuse swelling of a limb when the superficial and deep fascial layers are destroyed by infiltrative infection [9]. It is important to distinguish cellulitis from necrotizing fasciitis, as the treatment for cellulitis is not effective for necrotizing fasciitis.

While uterine tenderness, clinical fever, and vaginal discharge are common initial symptoms, our case illustrates that clinical diagnosis in the Emergency Room can be much more subtle, with a patient presenting with as vague a finding as unexplained tachycardia. As such, a high index of suspicion, especially among busy Emergency Room physicians, is critical for correct diagnosis and reduction of corresponding morbidity and mortality.

## Case presentation

A 37 year-old Caucasian female, G6P3033, post-partum day (PPD) #4, presented to labor and delivery as a transport from an outside hospital for symptoms of malaise, dyspnea, and tachycardia. The patient had had a vaginal delivery 4 days prior of a healthy male infant, complicated only by a midline episiotomy that was cut to expedite delivery in the setting of a non-reassuring fetal heart tone. Estimated blood loss was 300 cc. Her past medical history was significant for methylene tetrahydrofolate reductase heterozygous mutation, asthma, chickenpox in childhood, anxiety, and panic disorder. Her medications included ibuprofen and a prenatal vitamin. Her initial post-partum course was uncomplicated except for a new onset tachycardia of 120/min and transient shortness of breath of unknown etiology on PPD#1. This prompted a chest x-ray and pulmonary computed tomography (CT) scan, both of which were negative for an acute process or pulmonary embolus. Beside the new onset tachycardia, all other vital signs remained stable, the patient remained afebrile, and it was thus decided to send the patient home on PPD#2. A script for ibuprofen 600 mg q6 h was written for pain management.

Later that day, on PPD#2, the patient presented to an outside Emergency Room complaining of palpitations. She denied fevers, nausea, vomiting, abdominal pain, or purulent vaginal discharge. She complained of only minimal lochia, not excessive for a recent vaginal delivery. Vital signs were again stable, except for a tachycardia to 130/min. The patient remained afebrile. Hemoglobin and hematocrit were 9.5 g/dL and 28.5 % respectively, white blood cell count was 8.9 × 10^3^/μL and thyroid stimulating hormone was 1.28 UIU/mL. Electrocardiogram was performed showing a persistent sinus tachycardia. Echocardiography was within normal limits, showing a left ventricular ejection fraction of 60–65 %, normal left ventricular systolic function, no wall abnormalities, and no pericardial effusion. With no clear indication of infection, the etiology of the sinus tachycardia remained unknown and the patient was discharged from the ER on metoprolol 12.5 mg PO BID.

Two days later, on PPD#4, the patient presented to a second ER complaining of palpitations and dyspnea. She now complained of generalized body aches, and felt that she was too weak to walk. The patient again had tachycardia ranging from 120 to 135/min, with otherwise normal vital signs. Patient was again afebrile and denied uterine tenderness or purulent vaginal discharge. Chest CT and electrocardiogram were again normal with no acute processes. A right upper quadrant ultrasound and abdominal CT scan were significant for peri-hepatic fluid and mild splenomegaly but were otherwise unremarkable. At this time, the decision was made to transport the patient to the initial hospital where the delivery was performed, and where the first Emergency Room visit had occurred. Although slight suspicion of endometritis was raised, in the absence of a fever, uterine tenderness, or reported purulent discharge clinical suspicion remained low and there was no documentation of a pelvic or speculum examination.

Upon arrival at the new Emergency Room, the patient continued to complain of palpitations, shortness of breath, malaise, and generalized body aches. She felt unable to walk and developed diarrhea. Vital signs were again normal except for tachycardia into the 130’s/min. Patient remained afebrile with complete blood count showing a white blood cell count of 2.5 × 10^3^/μL, a creatinine of 1.0 mg/dL, and a blood urea nitrogen of 19 mg/dL. Three days early these values were white blood cell count of 9.6 × 10^3^/μL, creatinine of 0.8 mg/dL, and blood urea nitrogen of 6 mg/dL. Pelvic ultrasound showed no signs of retained products of conception. At this time, the decision was made to transport to our hospital, a tertiary care center for the region.

Shortly after arrival to our institution’s labor and delivery unit, a rapid response was called secondary to a pulse of 140/min, respiratory rate of 30/min, and blood pressure of 121/66 mmHg. Due to increasing concern for sepsis, the patient was sent to the Surgical Intensive Care Unit (SICU) and was started on piperacillin-tazobactam 3.375 g IV q 8 h. In the SICU a full physical exam was performed. On abdominal examination there was an approximately 2 cm × 2 cm area of erythema close to the umbilicus. A pelvic exam revealed diffuse erythema on the labia minora and labia majora. The cervix was noted to have two 0.5 cm white ulcerative lesions. There was foul smelling copious thick yellow liquid coming from the external cervical os. An endometrial curette was used to obtain an aerobic and anaerobic uterine culture. Bimanual exam revealed no retained products in the vagina or lower uterine segment. With close interaction between Obstetrics and Gynecology, Infectious Disease, and Surgical Intensive Care Unit services, a suspected diagnosis of Toxic Shock Syndrome (TTS) from probable Group A Streptococcus infection was made. This concern gained further validity when the patient provided information that both her husband and 2 year old child at home had streptococcus pyogenes (Strept Throat) infections over the previous 4 weeks. Results of blood cultures and uterine cultures later confirmed GAS infection. At this time the patient was started on intravenous immunoglobulin (IVIG) therapy. The recommendation from the Infectious Disease consultant was to achieve source control in this critically ill patient by performing a hysterectomy.

In the operating room, a laparotomy was performed, and a large boggy uterus, fallopian tubes, and ovaries were visualized. A decision was made to perform a total abdominal hysterectomy and bilateral salpingo-oophorectomy (TAHBSO) secondary to the diffuse nature of the infection. The proximal aspect of the vaginal cuff was also noted to be necrotic and this tissue was gradually debrided. Intra-operatively, Colorectal Surgery was consulted to inspect the integrity of the rectum. They performed a rigid proctoscopy which showed no visible rectal lesions. Exam of the perineum revealed no grossly necrotic areas in the vagina. Intraoperatively, the patient required 7U packed red blood cells, 5U fresh frozen plasma, 3U platelets, 10U cryoprecipitate. The patient remained intubated upon return to the SICU. She was started on ampicillin/sulbactam 3 g IV q6 h, and clindamycin 900 mg IV q8 h. Over the next twenty-four hours in the SICU, she required persistent use of norepinephrine and vasopressin, and developed hemorrhagic shock and disseminated intravascular coagulation. She returned to the OR for an exploratory laparotomy, where bleeding sites in the posterior cul-de-sac, and ovarian pedicles were again ligated and pelvic packing was placed. She required an additional 4U packed red blood cells, 4U fresh frozen plasma, 2U platelets, and 2U cryoprecipitate. She was taken to the operating room twenty-four hours later for reopening of the laparotomy site, perineal lavage, and removal of packing. The ovarian pedicles and vaginal cuff were both hemostatic, and cystoscopy showed bilateral ureteral jets. Forty-eight hours later she underwent one last abdominal washout procedure.

**Fig. 1:**
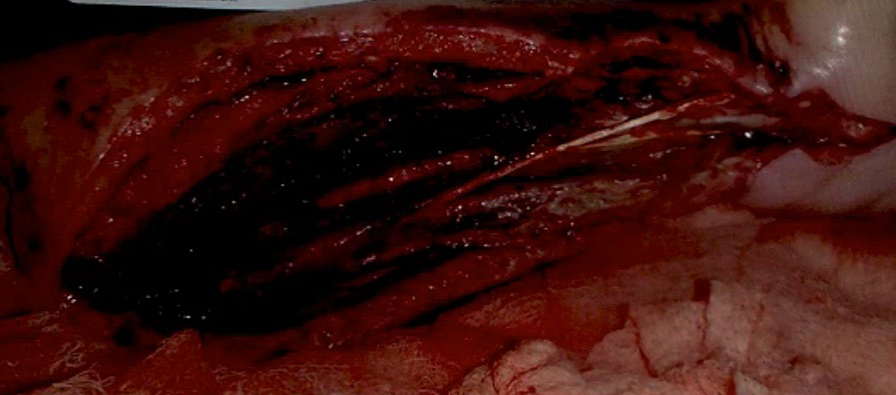
Left forearm intraoperative photograph #1. Left forearm status post decompressive fasciotomy of the flexor and extensor compartments as well as carpel tunnel release with median nerve neurolysis

**Fig. 2:**
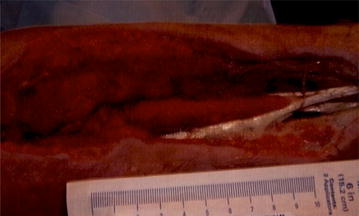
Left forearm intraoperative photograph #2. Left forearm status post decompressive fasciotomy of the flexor and extensor compartments as well as carpel tunnel release with median nerve neurolysis—zoomed view for scaling purposes

In the forty-eight hours after the TAHBSO, she developed an acute kidney injury with a creatinine spike of 1.8 mg/dL, urine output of approximately 30 cc/hr, and significant metabolic acidosis. She was started on continuous renal replacement therapy, and required this for the next 5 days. Vasopressors were utilized for a total of 4 days after the TAHBSO. Six days after the TAHBSO the patient was extubated in the SICU. Seven days after the TAHBSO, she began to develop compartment syndrome in her left forearm as well as paresthesias on the left median nerve distribution. She was consequently taken to the OR for a decompressive fasciotomy of the forearm, flexor and extensor compartments as well as carpel tunnel release with median nerve neurolysis (Figs. 1, 2). Finally, 13 days after the TAHBSO she was transferred out of the SICU to a floor bed where she remained for two more weeks before discharge. At discharge, the patient was to have extensive physical, occupational, and recreational therapy. Patient was feeling well upon discharge and had been following with multiple specialty clinics.

## Discussion

Puerperal infections can affect an estimated 5–10 % of pregnant women [1]. Pregnant and postpartum women have a 20-fold increased risk in invasive group A streptococcal (GAS) infection compared with non-pregnant women [2]. GAS infections occur predominantly in the postpartum period within the first 4 days, and maternal mortality is the highest within the first 2 days approaching 60 % when shock develops [6, 7]. As such, timely diagnosis and aggressive treatment of puerperal sepsis with antibiotics, fluids and source control is critical [10]. Since puerperal GAS infections can present with nonspecific symptoms, in our case with new onset persistent postpartum palpitations and tachycardia, healthcare providers, and especially Emergency Room physicians, must maintain a high level of suspicion.

Our case illustrates not only the importance of timely diagnosis and prompt treatment of puerperal GAS, but also how subtle initial symptomatology can be. This situation is complicated even further by the fact that these patients often present to busy emergency departments with these vague symptoms. These very same symptoms, including but not limited to fever, tachycardia, shortness of breath, myalgia, and weakness, are some of the most common complaints in all of medicine. The initial primary symptom in our case was persistent palpitations and tachycardia of unknown etiology, which pointed physicians away from GAS to consider predominantly cardiac and pulmonary etiologies. This complaint fueled an extensive cardiac and pulmonary evaluation at multiple facilities. On the contrary, the seemingly less obvious source of the complaint, an infectious etiology received less attention. Perhaps the seriousness of this situation could have been partially mitigated by increased early vigilance and cognizance to infectious etiology, specifically GAS symptomatology.

The purpose of this report is not to question the competency of the health care personnel who took care of the patient initially as the diagnosis was certainly quite challenging early on, with these challenges exacerbated exponentially with the hustle and bustle inherent to emergency rooms. However, much can be learned from this situation. In addition to gathering a history of present illness, a thorough gynecologic history, an antepartum history, intrapartum history, and postpartum history are all critical in the recent post partum patient. Such initial information helps to elicit any risk factors for postpartum infection. Recent lacerations, episiotomies, premature rupture of membranes, and mode of delivery, are all critical to know. Compromised mucosal or cutaneous barriers (e.g., episiotomy, vaginal mucosal tears, etc.) and a transiently more neutral vaginal pH after amniotic fluid release favoring organism growth are two factors that could increase the likelihood of GAS infection during this period [11]. Compromised immunity of pregnancy is undoubtedly also a risk factor. Knowledge of a previous upper respiratory tract GAS infection is also paramount as this can cause hematogenous seeding to the placenta and uterus. Asking about sick household contacts, specifically those recently diagnosed with strep throat (streptococcus pyogenes), is also critically important. Sick contacts may very well have been the source of infection in our case. Below are a few of the commonly presenting symptoms of GAS, although the presentation can vary greatly.

- Fever (greater than 38.5 °C within the first forty-eight hours postpartum and for greater than 4 h in duration)
  - Fever and pain in postpartum patients can be masked with liberal use of antipyretics such as acetaminophen and ibuprofen. This occurred in our case. Therefore, it is important to take this effect into account when evaluating postpartum patients who had a recent history of fevers, but are afebrile at presentation.
- Abdominal pain or tenderness
- Hypotension with or without tachycardia or leukocytosis
- Sudden onset of shock and organ dysfunction (renal failure, acute respiratory distress syndrome)

Streptococcal toxic shock syndrome is associated with higher mortality and presents with hypotension, tachycardia and leukocytosis. The diagnosis is made when the patient is hypotensive (systolic blood pressure ≤90 mmHg in adults) and streptococci are isolated from a previously sterile site, plus two or more of these findings [12]:

- Renal dysfunction (adults with normal renal function: creatinine ≥2 mg/dL)
- Coagulopathy
- Liver dysfunction (adults with normal liver function: transaminases or bilirubin ≥2× upper limit of normal)
- Acute respiratory distress syndrome
- Erythematous macular rash—may desquamate
- Soft tissue necrosis

Gram stain of tissues can be used for earlier diagnosis as it usually takes 8–24 h to recover organisms from blood cultures. However, if a case of puerperal sepsis is suspected in a postpartum patient, it is crucial to start antibiotics immediately to prevent life-threatening complications. Our case underscores the significance of not overlooking this diagnosis in a postpartum patient who may not necessarily fit the septic picture, as the consequences can be dire.

Screening all pregnant women for GAS colonization at the time of Group B Streptococcus screening may at first seem promising, but this too has many limitations, and is not routinely practiced in the United States. GAS colonization during pregnancy is exceedingly rare. For instance, one study in Vermont in which 35–37 week recto-vaginal swabs were cultured for GAS showed that only two individuals out of 6944 or 0.03 % had a positive GAS culture [13]. Further, in this study these two individuals did not receive antepartum treatment, yet neither of the mothers or infants became infected. Given the rarity of GAS colonization during pregnancy, compounded by the lack of clinical GAS infection even when a rare culture is positive argues against establishing routine GAS screening in pregnancy. A high index of suspicion in the postpartum patient with fevers, pain, abdominal tenderness, tachycardia, or hypotension, and comprehensive history taking likely represents a far more cost-effective approach to GAS identification and early therapeutic intervention.

We believe in the post partum patient with persistent palpitations and tachycardia presenting to the Emergency Room that a high level of suspicion needs to be maintained for pelvic infectious etiology even in the absence of reported pain, clinical fever, or vaginal discharge. This is supported by the known serious morbidity and mortality of GAS infection, as well as the time sensitive nature of diagnosis. Routine pelvic exam in the postpartum patient with tachycardia and palpitations, especially in the Emergency Department where these patients often present, may allow earlier diagnosis of this potentially highly morbid or even fatal infection.

## Conclusions

GAS infections are commonly suspected with fever, uterine tenderness, and vaginal discharge. The use of antipyretics may mask these symptoms. In our case, persistent palpitations and tachycardia in the post partum patient led to a missed diagnosis in numerous Emergency Rooms and increased morbidity from this time sensitive infection. A high index of suspicion is critical for diagnosis in the Emergency Room setting and early pelvic exam, with or without obstetric consultation, is likely prudent in the postpartum patient with unexplained palpitations and tachycardia.

## Consent

Written informed consent was obtained from the patient for publication of this case report and any accompanying images. A copy of the written consent is available for review by the Editor-in-Chief of this Journal.

## Abbreviations

GAS: Group A Streptococcus
PPD: Postpartum day
SICU: Surgical intensive care unit
TTS: Toxic shock syndrome
TAHBSO: Total abdominal hysterectomy bilateral salpingo-oophorectomy
